# In Reply to: “Response to intra-arterial chemotherapy in patients with adenoid cystic carcinoma of lacrimal gland”

**DOI:** 10.1093/oncolo/oyag087

**Published:** 2026-04-07

**Authors:** Philippos Apolinario Costa, David T Tse, Pasquale Benedetto

**Affiliations:** Department of Medicine, Division of Oncology, Yale University, New Haven, CT, United States; Department of Ophthalmology, Bascom Palmer Eye Institute, University of Miami Miller School of Medicine, Miami, FL, United States; Department of Medicine, Division of Oncology, Sylvester Comprehensive Cancer Center, University of Miami Miller School of Medicine, Miami, FL, United States

We appreciate Dr. Esmaeli’s thoughtful engagement with our work^1^ and the opportunity to further clarify aspects of our expanded experience with neoadjuvant intra-arterial chemotherapy (IAC) for lacrimal gland adenoid cystic carcinoma (ACC).

With respect to the observed difference in radiographic response rates between the original cohort and the additional 16 patients, there were no changes in technique, catheterization approach, drugs administered, dosing, or route of delivery over time. The IAC protocol remained consistent across both cohorts. The reduction in radiographic response rate in the expanded series therefore does not reflect a modification in treatment delivery. As our experience accrued and the case mix broadened, some variability in response is expected. Potential explanations include tumor biologic heterogeneity, differences in baseline tumor characteristics such as grade and infiltrative pattern, and the inherent variability of retrospective radiographic assessment.

The vascular supply of lacrimal gland tumors may plausibly influence treatment efficacy, particularly given the variable contributions of branches from the external and internal carotid systems. Tumors predominantly supplied by internal carotid branches could theoretically receive less effective drug delivery when infusion is performed through external carotid branches. However, in this retrospective series, preoperative angiography or MRA was not systematically performed or recorded in a manner that permits reliable correlation between vascular anatomy and radiographic response. This represents an important area for prospective data collection.

Regarding response assessment, two patients (Patients 25 and 26) were designated as not assessable because they underwent unplanned R1 resections at outside institutions immediately before referral. IAC was administered in the re-resection setting, and there was no measurable radiographic disease at the time of treatment to permit standard response evaluation. Both patients are currently without evidence of disease despite their initial positive-margin resections. Among evaluable patients, three individuals (Patients 20, 33, and 35) did not demonstrate radiographic tumor shrinkage following IAC. Two had stable disease as their best response, and one had progression. One of these patients received only a single cycle due to a reversible acute kidney injury and did not complete protocol therapy. It is also important to emphasize that the potential benefits of neoadjuvant chemotherapy extend beyond radiographic shrinkage alone, as pathologic treatment effect and potential reduction in micrometastatic disease are clinically meaningful endpoints not fully captured by imaging-based response criteria.

With respect to staging, we intentionally used the 7th edition of the AJCC system to maintain consistency with our original publication. We recognize that the 8th edition includes important refinements. To facilitate reclassification, we have provided raw tumor size data and information regarding invasion of adjacent structures (Table 1). However, precise reassignment according to the 8th edition would require detailed delineation of specific structures involved (e.g. bone, optic nerve, brain), which was not uniformly documented in a manner sufficient for accurate retrospective restaging and would require a dedicated re-review study.

**Table 1.** Tumor size and adjacent structure invasion.

ID	Size (cm)	Invasion of adjacent structures
**20**	1.5	No
**21**	1.8	No
**22**	4	No
**23**	2.6	Yes
**24**	2.7	Yes
**25**	0.8	No
**26**	2	No
**27**	3	No
**28**	2.9	No
**29**	3.8	No
**30**	5.2	No
**31**	1.5	No
**32**	3.3	No
**33**	2.2	No
**34**	4.3	No
**35**	4.3	Yes
**36**	3.5	Yes
**37**	3.1	No
**38**	2.9	No
**39**	3.4	No
**40**	2.9	Yes
**41**	2.2	No
**42**	2	No

We agree that the SEER database has important limitations, including a lack of granularity regarding AJCC edition–specific staging and the absence of detailed treatment information. Historical survival estimates derived from SEER may not fully reflect contemporary outcomes. We also acknowledge that more recent series of eye-sparing surgery followed by radiation therapy report outcomes that appear improved relative to older historical controls. However, such series are inherently subject to substantial selection bias, as eye-sparing approaches are typically offered to patients with smaller, more favorably located tumors. In contrast, population-based registries such as SEER likely capture a broader and more heterogeneous population, including patients with advanced disease who may not be candidates for eye-sparing strategies. An ideal comparison would require a prospective randomized study, which is challenging given the rarity of lacrimal gland ACC, funding constraints, and strong patient preferences—particularly reluctance to be randomized to surgery alone.

Finally, regarding patients with T1 tumors, four individuals in our cohort (Patients 20, 21, 25, and 31) were staged as T1. These patients were not uniformly treated with orbital exenteration, and not all patients in the series—including those with higher T-category disease—underwent exenteration. Two of the T1 patients (25 and 31) were complex salvage cases following prior eye-sparing surgery at outside institutions. Only one T1 patient (Patient 21) underwent orbital exenteration. As noted above, published eye-sparing surgery series are highly selected, which likely contributes to favorable reported outcomes. Similar survival rates between those series and broader, more inclusive cohorts such as ours should therefore be interpreted cautiously and do not imply superiority of the eye-sparing approach. Selection bias in surgical oncology tends to inflate outcomes in carefully selected populations; correcting for that bias would not be expected to further improve those results. The implication that correcting for selection bias would yield even better outcomes in eye-sparing series reflects a misunderstanding of how such bias operates in surgical oncology cohorts.
